# Supplementary material for: In vitro proteasome processing of neo-splicetopes does not predict their presentation in vivo
Source: eLife. 2021 Apr 20;10:e62019. doi: 10.7554/eLife.62019 (PMC8154032; doi:10.7554/eLife.62019)
Supplement: Supplementary file 1. [file elife-62019-supp1.docx]

**HLA-ABC haplotypes of BLCLs and tumor cell lines SW480 and SW620**

|  | **HLA-A** | | **HLA-B** | | **HLA-C** | |
| --- | --- | --- | --- | --- | --- | --- |
| **SW620** | 02:01 | 24:01 | 07:13 | 37:04 | 07:04 | 07:04 |
| **SW480** | 02:01 | 24:02 | 07:02 | 15:18 | 07:04 | 07:04 |
| **LCLW03** | 02:01 | 23:01 | 15:01 | 58:01 | 03:04 | 07:01 |
| **JY** | 02:01 |  | 07:02:01 |  | 07:02:01:01 |  |
| **KLO** | 02:08 | 01:01:01:01 | 08:01:01 | 50:01:01 | 07:01:01:01 | 06:02:01:02 |
| **WT49** | 02:05:01 |  | 58:01:01 |  | 07:18 |  |
| **BM14** | 03:01 |  | 07:02 |  | 07:02 |  |
| **SA** | 24:02:01:01 |  | 07:02:01 |  | 07:02 |  |
| **VAVY** | 01:01 |  | 08:01 |  | 07:01 |  |
