# Supplementary material for: In vitro proteasome processing of neo-splicetopes does not predict their presentation in vivo
Source: eLife. 2021 Apr 20;10:e62019. doi: 10.7554/eLife.62019 (PMC8154032; doi:10.7554/eLife.62019)
Supplement: Supplementary file 2. [file elife-62019-supp2.docx]

**Faulty peptides identified within the KRAS^G12V^** **polypeptide substrates**

| **Faulty peptides identified within the KRAS^G12V^_2-14_** **polypeptide substrate**  **T E Y K L V V _ G A V G V 5-8/10-14 sp1**  **E Y K L V V _ G A V G V**  **T E Y K L V V V G _ V G V 5-9/11-14 sp2**  **T E Y K L V V V _ A V G V 5-10/12-14 sp4**  **Faulty peptides identified within the KRAS^G12V^_2-21_** **polypeptide substrate**  **T E Y K L V V _ G A V G V V G S K S A L T I 5-8/10-14 sp1**  **K L V V _ G A V G V V G S K S A L T I**  **K L V V _ G A V G V V G S K S A**  **T E Y K L V V V G _ V G V V G S K S A L T I 5-10/12-14 sp4**  **K L V V V G _ V G V V G S K S A**  **K L V V V G _ V G V V G S K S A L** |
| --- |
